# Does Preoperative Acute Pancreatitis Inevitably Delay Pancreatoduodenectomy in Patients with Periampullary Tumors?

**DOI:** 10.3390/cancers13246289

**Published:** 2021-12-15

**Authors:** So Jeong Yoon, Okjoo Lee, Ji Hye Jung, Sang Hyun Shin, Jin Seok Heo, In Woong Han

**Affiliations:** Division of Hepatobiliary-Pancreatic Surgery, Department of Surgery, Samsung Medical Center, Sungkyunkwan University School of Medicine, Seoul 06351, Korea; wooyabi@gmail.com (S.J.Y.); okjoo7.lee@samsung.com (O.L.); sog-hei@daum.net (J.H.J.); sanghyun80.shin@samsung.com (S.H.S.); jsheo@skku.edu (J.S.H.)

**Keywords:** preoperative acute pancreatitis, periampullary tumors, pancreatoduodenectomy, postoperative complication, endoscopic retrograde cholangiopancreatography

## Abstract

**Simple Summary:**

Acute pancreatitis can occur preoperatively in patients with periampullary tumors and cause technical difficulties in performing pancreatoduodenectomy. The aim of this retrospective study was to investigate how preoperative acute pancreatitis would affect postoperative outcomes and to identify the optimal timing of surgery. There were more patients with operation failure (only exploration or unintended total pancreatectomy) in patients with pancreatitis, but no difference was found in rates of other complications. Moreover, when stratified by the timing of surgery, the surgical outcomes did not differ between the patients with and without pancreatitis. The results imply that in terms of surgical complications, pancreatoduodenectomy could be safely performed in patients with preoperative pancreatitis. Further research is necessary to identify safe conditions and proper timing of surgery for patients with preoperative pancreatitis.

**Abstract:**

Preoperative acute pancreatitis (PAP) in patients with periampullary tumor can cause technical difficulties when performing pancreatoduodenectomy (PD) but perioperative risks of PAP remain unclear. The purpose of this study was to investigate the impact of PAP on surgical outcomes and determine the optimal timing of PD. Patients undergoing surgery for periampullary tumors between 2009 and 2018 were included. Simple random sampling (1:4) was performed to compare outcomes between the PAP group and the control group. Operative failure was defined as exploration-only or unwanted total pancreatectomy. The rate of operative failure was higher in the PAP group than in the control group (6.6% vs. 0%, *p* < 0.001). There was no significant difference in postoperative outcomes including complications or in-hospital mortality between the two groups. Surgical outcomes were compared after dividing PAP groups by intervals (2, 3, or 4 weeks) between the onset of PAP and surgery, and there were no differences between the groups. In conclusion, in spite of the increased risk of operation failure, PD could be performed in PAP patients at comparable rates of postoperative complications. Further study is needed to select patients with PAP in proper conditions for performing PD.

## 1. Introduction

Pancreatoduodenectomy (PD) is a surgical procedure that is performed in patients with periampullary tumors for curative resection. PD is one of the most complex surgeries with mortality up to 2% and morbidity up to 40% even in high-volume centers [1]. Major complications after PD include post-pancreatectomy hemorrhage, postoperative pancreatic fistula (POPF), and intra-abdominal abscess. These complications can delay the time of adjuvant therapy for patients who should receive it [2].

One condition that surgeons should take into consideration before performing PD is preoperative acute pancreatitis (PAP). It is known that acute pancreatitis (AP) might be recognized as a presentation, sometimes initially, in patients with pancreatic cancer [3]. AP can also occur as a complication after endoscopic retrograde cholangiopancreatography (ERCP) for tissue confirmation or preoperative biliary drainage in patients with obstructive jaundice. It was reported that the overall incidence of post-ERCP pancreatitis was 9.7%, with half of patients suffering from moderate to severe pancreatitis [4]. 

Although PAP might cause technical challenges, there are only limited data available regarding the impact and risks of PAP and the optimal timing of PD in patients with PAP. Thus, the aim of this study was to investigate the impact of PAP on postoperative outcomes and determine the optimal timing of PD in patients with PAP. This article is presented in accordance with the STROBE reporting checklist.

## 2. Materials and Methods

### 2.1. Patient Database

A total of 1328 patients underwent surgical treatment for periampullary tumors (tumors of the duodenum, pancreas head, distal bile duct, or ampulla of Vater) in the Samsung Medical center between January 2009 and December 2018. Their preoperative laboratory testing, image scans, operation records, pathology reports, and postoperative outcomes were retrospectively reviewed. This study was approved by the Institutional Review Board of Samsung Medical Center (Seoul, Korea, approval no. 2020-11-012). The Institutional Review Board waived the need for written informed consent from the patients since the research involved no more than minimal risk to subjects, and there was no reason to assume rejection of agreement.

### 2.2. Diagnosis and Management of Acute Pancreatitis

Patients who were diagnosed with PAP within three months before surgery were included in the PAP group. Diagnosis and grading of PAP were done according to the 2012 revised Atlanta criteria [5]. The diagnosis required at least two of the following three features: (1) abdominal pain consistent with acute pancreatitis (acute onset of a persistent, severe, epigastric pain often radiating to the back); (2) serum lipase activity (or amylase activity) at least three times greater than the upper limit of normal; and (3) characteristic findings of acute pancreatitis on contrast-enhanced computed tomography (CT), magnetic resonance imaging (MRI) or transabdominal ultrasonography. If patients had organ failure or local complications, they were categorized as having moderate to severe PAP. In terms of the etiology, it was regarded as ERCP-related if symptoms occurred within a week after endoscopic procedures. When patients had no history of endoscopic examinations but intermittent abdominal pain with elevated serum amylase/lipase at the time of diagnosis, it was considered tumor related.

For patients with mild pancreatitis without organ failure, conservative measures such as intravenous hydration were used. Patients with organ dysfunctions were admitted to intensive care units until acute conditions were resolved. The timing of surgery was determined at the discretion of surgeons, but either follow-up CT scans or laboratory tests including serum amylase/lipase were performed within a week before surgery, in order to confirm improved inflammatory status and resolution of organ dysfunctions.

### 2.3. Postoperative Outcomes

Surgical outcomes were recorded by attending physicians involved in the post-operative management of patients. Given that oncologic surgery should focus on curative resection and safety, operation failure included cases in which pancreatic resection could not be done after exploratory laparotomy (exploration-only) or total pancreatectomies were unavoidable due to pancreatitis. Surgeons decided to perform total pancreatectomies when it was impossible to transect the pancreatic neck or to perform pancreatojejunostomy due to severe adhesion and inflammation. The severity of complications was graded using the Clavien-Dindo (CD) classification [6]. POPF was diagnosed and graded according to the 2016 International Study Group for Pancreatic Fistula definition and grading [7]. Mortality and readmission rates within 90 days after discharge were reviewed.

### 2.4. Statistical Analysis

For comparison with patients of the PAP group, a simple random sampling of 4:1 was performed to select patients without PAP (the control group). Considering disease heterogeneity, the ratio of pancreatic to non-pancreatic tumors was matched between the two groups. Comparisons of preoperative clinical characteristics, intraoperative findings, and postoperative outcomes were conducted using Student’s *t*-test, chi-squared test, and Fisher’s exact test. Missing values were excluded from the analysis. Variables with *p*-values less than 0.05 were regarded as statistically significant. All statistical analyses were performed using IBM SPSS version 26 (SPSS Inc., Chicago, IL, USA).

## 3. Results

Among all patients who underwent PD, 91 were diagnosed with PAP. Accordingly, 364 patients without PAP were randomly assigned to the control group. Clinicopathological characteristics of the two groups are shown in Table 1. There were more patients with American Society of Anesthesiologists (ASA) scores of III to IV in the PAP group than in the control group (13.2% vs. 6.0%, *p* = 0.020). In the PAP group, 27.5% of patients had moderate to severe PAP. PAP occurred secondary to periampullary masses (54.9%) or following ERCP (40.7%). Intraoperatively, the rate of operation failure was 6.6% in the PAP group and 0% in the control group (*p* = 0.019). More patients in the PAP group had hard pancreatic texture than in the control group (63.1% vs. 33.7%, *p* < 0.001).

Table 2 shows the comparison of surgical outcomes between the two groups. There were no statistically significant differences in rates of complications that were more severe than CD classification III and POPF between the two groups. The length of hospital stay was comparable. Mortality and re-admission rates did not differ significantly either between the two groups.

To investigate the impact of waiting time for operation, comparisons of surgical outcomes were performed after stratification of the PAP group by the interval (2, 3, and 4 weeks) from diagnosis of PAP to surgery (Table 3). When levels of maximal preoperative C-reactive protein (CRP) and levels immediately before surgery were compared, there were no statistically significant differences among different interval groups. The rate of operation failure was not significantly different according to waiting time for operation. Rates of postoperative complications were also comparable among different interval groups. 

**Figure 1:**
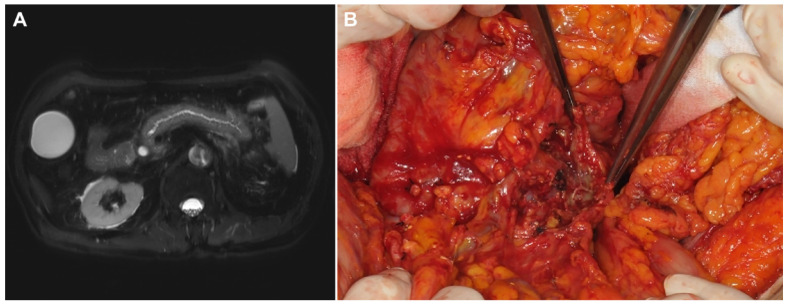
Preoperative image and operative findings of a PAP patient with an operation failure. (**A**) Preoperative magnetic resonance image (MRI) showing peripancreatic infiltration suggestive of acute pancreatitis after endoscopic retrograde cholangiopancreatography (ERCP). (**B**) Operation findings of the patient showing necrotic change in pancreas at the cut surface which led to total pancreatectomy.

**Figure 2:**
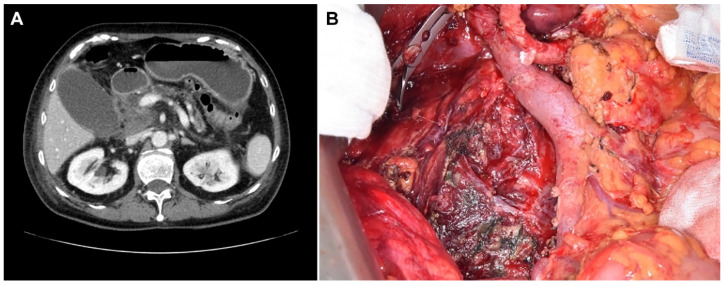
Preoperative image and operative findings of a PAP patient with a successful pancreatoduodenectomy. (**A**) Preoperative computed tomography (CT) image showing post-ERCP pancreatitis. (**B**) Operation findings of the patient after pancreatoduodenectomy showing debris of necrotic tissues.

Six patients in the PAP group experienced operative failure (Table 4), with two of them undergoing exploration only and the other four undergoing unintended total pancreatectomies. Of these six patients, three had only mild pancreatitis according to the revised Atlanta criteria. However, necrotic changes of the entire pancreas were found during the operation which led to operation failure. There was no 90-day mortality case in these six patients. Among other patients in the PAP group who underwent pancreatectomies as planned, about one-half of them had more severe pancreatitis than expected preoperatively. Figure 1 shows an operation failure case in a patient with PAP. Figure 2 shows an exemplary case of a patient with PAP after ERCP.

## 4. Discussion

PAP is not a rare condition in patients with periampullary tumors who are waiting for surgeries. In the absence of sufficient data on postoperative outcomes of patients with PAP, the current study aimed to identify the clinical impact of PAP and the optimal timing of PD in patients with PAP. Although the rate of operation failure was significantly higher in the PAP group than in the control group, there was no difference in adverse surgical outcomes between the two groups. The time to surgery from the diagnosis of PAP did not affect the postoperative outcomes.

From various degrees of severity of the patients with PAP and operation failure, it is suggested that preoperative clinical manifestation and severity might not always correspond to operative findings. A previous retrospective study with ten PAP patients has implied that high preoperative CRP levels could indicate more severe pancreatitis which might lead to total pancreatectomy [8]. In our study, maximal and immediate preoperative levels of CRP were not associated with either preoperative severity of pancreatitis or intervals between diagnosis and surgery. Further studies are necessary to identify preoperative factors capable of predicting the severity of inflammation in the operative field.

In terms of causes of PAP, 54.9% of the PAP group had idiopathic acute pancreatitis not related to common etiologies (such as alcohol, gallstones) or preoperative endoscopic procedures. Most of these patients had a history of recurrent abdominal pain before they visited medical institutions. In addition, most of them were diagnosed with AP associated with periampullary tumors in imaging evaluation or laboratory tests. It has been known that AP may develop in patients with pancreatic cancer, although a relatively low number of individuals present symptoms [3,9]. One study including 45 patients with AP reported that the time between the onset of AP and the diagnosis of tumor ranged from 1 to 52 weeks and that patients had an average of two episodes [3]. In terms of severity, 75% of patients with mass-related PAP in our cohort presented mild AP, comparable with findings of previous studies [3,10]. Considering recurrent episodes of mild AP in these patients, there would be no benefit of delaying the operation. 

As to preoperative ERCP, it is often performed for tissue sampling and biliary drainage in patients with periampullary tumors. In our institution, decisions on ERCP were made after discussion between endoscopists and surgeons, considering the risk and benefits of procedures. As shown in Table 1, most of the patients with preoperative ERCP needed intervention for biliary drainage. The rate of post-ERCP pancreatitis (PEP) could increase up to 30%, after interventional ERCP [11]. There have been several attempts to decrease the incidence and severity, with some guidelines recommending prophylactic measures [12,13]. In a previous systematic review of 12 studies, female gender, previous PEP, sphincterotomy, and sphincter of Oddi dysfunction were risk factors for PEP [14]. If PEP involves sepsis with infected pancreatic necrosis, surgical plans for patients might be altered. Recently, magnetic resonance cholangiopancreatography (MRCP) and endoscopic ultrasonography (EUS) are frequently used as diagnostic imaging modalities for periampullary diseases. Previous studies reported that these tools are as sensitive as ERCP for detecting periampullary carcinoma and that they may prevent unnecessary explorations of bile or pancreatic duct with the endoscopic procedure [15,16]. Regarding preoperative biliary drainage (PBD), several previous retrospective studies showed that it could reduce postoperative morbidity and mortality [17,18]. However, a few other studies including a randomized control trial reported that routine PBD increased the rate of postoperative complications [19,20]. Although there is no consensus yet on the severity of jaundice that requires PBD, a recent study suggested that PBD might have a beneficial role in patients with a bilirubin level of 250 µmol/L (14.6 mg/dL) or higher [21]. In this regard, interventional ERCP should be performed selectively in patients when tissue confirmation or PBD is unavoidable. 

It is notable that our findings are inconsistent with those of previous studies. One retrospective study including six patients with severe PAP suggested that a waiting time of at least three months was necessary to ensure that inflammation was localized to the peripancreatic area [22]. The authors argued that severe PAP could lead to the development of POPF which prolonged hospital stay. Chen et al. [23] included 38 PAP patients and reported that PAP significantly increased the rate of severe complications. In our study, on the contrary, the mean waiting time was less than a month. However, rates of curative resection and complications did not differ significantly between the PAP group and the control group. An implication of these results is that PD could be performed safely without delay in patients with PAP considering that a delay in treatment might lead to a poor prognosis of patients with periampullary cancers. 

This study has several limitations. First, since this was a single-center retrospective study, it had numerous biases. The patients with PAP were identified by retrospective chart review and disease evaluation including the need for biliary intervention was mostly done in the department of gastroenterology. Moreover, there was no principle on how to manage PAP patients. Since recommendations for the initial treatment of acute pancreatitis have been changed during the study period, the management was not standardized among physicians. Furthermore, an analysis of infection was not performed in the present study. Considering that infections could significantly affect the condition of patients with PAP, it would be important to include data on infection or sepsis in a further prospective study. Second, the timing of the operation was inconsistent among surgeons. This might have caused selection and recall biases. In addition, owing to disease heterogeneity, long-term oncologic outcomes related to the timing of surgery could not be investigated. The oncologic effects of PAP itself and delay in treatment should be examined in future studies. 

Despite these shortcomings, the main strength of the present study was that it included a large number of PAP patients and revealed that PD could be safely performed in patients with PAP without increasing risks of postoperative complications. Further well-designed prospective studies are needed to determine clinical implications and effects of PAP on surgical outcomes in patients with periampullary tumors. 

## 5. Conclusions

We investigated the impact of PAP on surgical outcomes after PD. It was identified that in spite of a probability of operation failure, PAP did not increase postoperative complications. Further studies are necessary to identify appropriate timing and conditions of PAP patients for undergoing PD.

## Figures and Tables

**Table 1:** Comparisons of clinicopathological characteristics and intraoperative findings between patients in the PAP group and the control group.

Variables	PAP Group (*n =* 91)	Control Group (*n =* 364)	*p*-Value
Age, mean	62.4 (±10.4)	63.5 (±10.1)	0.354
Sex			0.164
Male	61 (67.0%)	215 (59.1%)	
Female	30 (33.0%)	149 (40.9%)	
BMI (kg/m^2^), mean	23.3 (±2.9)	23.2 (±3.2)	0.968
ASA score			0.020
I–II	79 (86.8%)	342 (94.0%)	
III–IV	12 (13.2%)	22 (6.0%)	
Neoadjuvant therapy, Yes	2 (2.2%)	10 (2.7%)	>0.99
Preop. ERCP, Yes	48 (52.7%)	168 (46.2%)	0.260
Preop. Endoscopic drainage, Yes	42 (46.2%)	143 (39.3%)	0.233
Preop. Acute pancreatitis		N/A	
Mild	66 (72.5%)		
Moderate to Severe	25 (27.5%)		
Causes of pancreatitis		N/A	
Mass	50 (54.9%)		
Endoscopic procedure	37 (40.7%)		
Unknown	4 (4.4%)		
Time to surgery, mean (days)	26.5 (±21.3)	N/A	
Pathology			>0.99
Pancreas tumors	63 (69.2%)	252 (69.2%)	
Others	28 (30.8%)	112 (30.8%)	
Type of Surgery			0.019
Pancreatoduodenectomy	83 (91.2%)	349 (95.9%)	
Total pancreatectomy	6 (6.6%)	15 (4.1%)	
Exploration only	2 (2.2%)	0 (0%)	
Operation failure	6 (6.6%)	0 (0%)	<0.001
Operation time (min), mean	323.1 (±65.0)	323.3 (±74.0)	0.980
Pancreas texture			<0.001
Soft	13 (15.5%)	117 (35.2%)	
Moderate	18 (21.4%)	103 (31.0%)	
Hard	53 (63.1%)	112 (33.7%)	
Intraop. Transfusion, Yes	10 (11.0%)	31 (8.5%)	0.461
R0 resection	74 (81.3%)	296 (81.3%)	>0.99

Abbreviations: PAP—preoperative acute pancreatitis; BMI—body mass index; ASA—American society of anesthesiologist; Preop.—preoperative; ERCP—endoscopic retrograde cholangiopancreatography; Intraop.—intraoperative.

**Table 2:** Comparisons of surgical outcomes between patients in the PAP group and the control group.

Variables	PAP Group (*n =* 91)	Control Group (*n =* 364)	*p*-Value
C-D grade ≥ III complications	16 (17.6%)	79 (21.7%)	0.387
POPF	32 (35.2%)	155 (42.6%)	0.198
CR-POPF	5 (5.5%)	38 (10.4%)	0.149
Postoperative hemorrhage	5 (5.5%)	24 (6.6%)	0.701
Intra-abdominal infection	8 (8.8%)	16 (4.4%)	0.113
Biliary fistula	1 (1.1%)	2 (0.5%)	0.489
Chyle leak	7 (7.7%)	29 (8.0%)	0.931
Surgical site infection	2 (2.2%)	22 (6.0%)	0.191
Delayed gastric emptying	8 (8.8%)	31 (8.5%)	0.933
Length of stay (postoperative days)	13.0 (±7.6)	14.0 (±8.6)	0.353
90-day mortality	1 (1.1%)	8 (2.2%)	0.695
90-day re-admission	11 (12.1%)	38 (10.4%)	0.650

Abbreviations: PAP—preoperative acute pancreatitis; C-D—Clavien-Dindo; POPF—postoperative pancreatic fistula; CR-POPF—clinically relevant postoperative pancreatic fistula.

**Table 3:** Comparisons of surgical outcomes between patient groups stratified by the time to surgery in patients with PAP (*n =* 91).

Variables	Within 2 Weeks (*n =* 32)	After 2 Weeks (*n =* 59)	*p*-Value	Within 3 Weeks (*n =* 51)	After 3 Weeks (*n =* 40)	*p*-Value	Within 4 Weeks (*n =* 62)	After 4 Weeks (*n =* 29)	*p*-Value
Moderate to severe pancreatitis	7 (21.9%)	18 (30.5%)	0.378	12 (23.5%)	13 (32.5%)	0.341	14 (22.6%)	11 (37.9%)	0.126
Preop. ERCP	17 (53.1%)	31 (52.5%)	0.958	28 (54.9%)	20 (50.0%)	0.642	35 (56.5%)	13 (44.8%)	0.301
CRP, preop. max.	4.6	5.1	0.753	4.8	4.9	0.931	4.2	6.6	0.158
immediate preop.	2.2	2.1	0.869	2.3	1.9	0.576	2.1	2.2	0.910
Pathology, pancreatic tumors	22 (68.8%)	41 (69.5%)	0.942	35 (68.6%)	28 (70.0%)	0.888	43 (69.4%)	20 (69.0%)	0.970
Operation time (mins)	324.0	315.9	0.603	325.1	310.6	0.335	322.4	310.9	0.473
Pancreatic texture, soft	4 (12.5%)	9 (15.3%)	>0.99	8 (15.7%)	5 (12.5%)	0.666	8 (12.9%)	5 (17.2%)	0.749
Operation failure	1 (3.1%)	5 (8.5%)	0.419	4 (7.8%)	2 (5.0%)	0.691	4 (6.5%)	2 (6.9%)	>0.99
Exploration only	0 (0%)	2 (3.4%)	0.539	1 (2.0%)	1 (2.5%)	>0.99	1 (1.6%)	1 (3.4%)	0.538
Conversion to TP	1 (3.1%)	3 (5.1%)	>0.99	3 (5.9%)	1 (2.5%)	0.628	3 (4.8%)	1 (3.4%)	>0.99
C-D grade ≥ III complications	4 (12.5%)	12 (20.3%)	0.348	8 (15.7%)	8 (20.0%)	0.592	11 (17.7%)	5 (17.2%)	0.953
CR-POPF	1 (3.1%)	4 (6.8%)	0.653	1 (2.0%)	4 (10.0%)	0.165	4 (6.5%)	1 (3.4%)	>0.99
Postoperative hemorrhage	1 (3.1%)	4 (6.8%)	0.653	4 (7.8%)	1 (2.5%)	0.380	4 (6.5%)	1 (3.4%)	>0.99
Intra-abdominal infection	5 (15.6%)	5 (8.5%)	0.313	7 (13.7%)	3 (7.5%)	0.503	8 (12.9%)	2 (6.9%)	0.493
Biliary fistula	0 (0%)	1 (1.7%)	>0.99	1 (2.0%)	0 (0%)	>0.99	1 (1.6%)	0 (0%)	>0.99
Chyle leak	4 (12.5%)	3 (5.1%)	0.236	4 (7.8%)	3 (7.5%)	>0.99	5 (8.1%)	2 (6.9%)	>0.99
Surgical site infection	0 (0%)	2 (3.4%)	0.539	0 (0%)	2 (5.0%)	0.190	0 (0%)	2 (6.9%)	0.099
Delayed gastric emptying	4 (12.5%)	2 (6.8%)	0.445	5 (9.8%)	3 (7.5%)	>0.99	7 (11.3%)	1 (3.4%)	0.428
Length of stay (days)	13.4	12.8	0.737	13.9	11.9	0.227	13.7	11.5	0.179
90-day re-admission	3 (9.4%)	8 (13.6%)	0.741	6 (11.8%)	5 (12.5%)	>0.99	7 (11.3%)	4 (13.8%)	0.739

Abbreviations: PAP—preoperative acute pancreatitis; Preop.—preoperative; ERCP—endoscopic retrograde cholangiopancreatography; Max.—maximal; TP—total pancreatectomy; C-D—Clavien-Dindo; CR-POPF—clinically relevant postoperative pancreatic fistula.

**Table 4:** Clinicopathologic characteristics and operative findings of patients with operation failure (*n =* 6).

No.	Age/Sex	Tumor Location	Cause of PAP	Severity	Preop. Max. Serum Amy/Lip (U/L)	Preop. Max. CRP (mg/dL)	Time to OP (days)	OP Name	Field Findings
1	59/F	AoV	mass	Moderate	-	-	45	Exploration only	Adhesion around the pancreas Unable to approach pancreas
2	58/M	Bile duct	ERCP	Severe	527/921	0.07	90	Total pancreatectomy	Severe necrotizing pancreatitis Hard to identify SMV
3	72/F	Bile duct	ERCP	Mild	1684/3827	9.54	7	Total pancreatectomy	Necrotic change of the whole pancreas Unable to perform PJ anastomosis
4	69/M	Bile duct	ERCP	Mod	1260/3703	19.39	15	Total pancreatectomy	Necrotic change of the whole pancreas Unable to perform PJ anastomosis
5	77/M	Bile duct	mass	Mild	100/382	0.92	20	Exploration only	Necrotic change of the whole pancreas Bleeding tendency with friable tissue
6	61/M	Pancreas	mass	Mild	676/1713	8.63	19	Total pancreatectomy	Necrotic change of the whole pancreas Unable to perform PJ anastomosis

Abbreviations: PAP—preoperative acute pancreatitis; Preop.—preoperative; Max.—maximal; amy—amylase; lip—lipase; OP—operation; F—female; M—male; AoV—ampulla of Vater; ERCP—endoscopic retrograde cholangiopancreatography; SMV—superior mesenteric vein; PJ—pancreaticojejunostomy.

## Data Availability

The data presented in this study are available in this article.
